# Evaluating the effectiveness of sociotherapy following psychiatric hospitalization: a target trial emulation protocol using German statutory health insurance data

**DOI:** 10.1186/s12913-025-13137-2

**Published:** 2025-07-31

**Authors:** Raphael Kohl, Sophia Zander, Christian Hering, Udo Schneider, Stefanie Schreiter, Julie L. O’Sullivan

**Affiliations:** 1 Charité– Universitätsmedizin Berlin, corporate member of Freie Universität Berlin and Humboldt-Universität Zu Berlin, Institute of Medical Sociology and Rehabilitation Science, Charitéplatz 1, 10117 Berlin, Germany; 2 Techniker Krankenkasse, Hamburg, Germany; 3 Department of Psychiatry and Neurosciences, Charité– Universitätsmedizin Berlin, corporate member of Freie Universität Berlin and Humboldt-Universität Zu Berlin, Charité Campus Mitte, Charitéplatz 1, 10117 Berlin, Germany

**Keywords:** Sociotherapy, Case management, Discharge management, Transitional intervention, Psychiatric hospital treatment, Target trial emulation

## Abstract

**Background:**

The German health care system maintains a strict division between inpatient and outpatient care, posing challenges for the continuity of care. Discharge management facilitates the transition from inpatient to outpatient settings. For psychiatric patients, sociotherapy is one transitional intervention that can be prescribed to inpatients at discharge and then provided in the outpatient setting. As a form of case management, it aims to motivate patients, coordinate care among providers, and ultimately reduce psychiatric hospital readmissions. However, the effectiveness of sociotherapy remains unclear due to a lack of randomized trials. This protocol describes an observational study investigating the causal impact of sociotherapy on psychiatric readmission rates.

**Methods:**

We will use claims data from the largest statutory health insurance provider in Germany to evaluate the effect of sociotherapy as a discharge management measure on 30-day readmissions among patients who received psychiatric inpatient treatment. To reduce potential bias in this observational study we will use the target trial emulation framework. Quasi-randomization will be achieved through Propensity Score based methods, controlling for characteristics of the index event, patients' medical history, and relevant sociodemographic factors. Intention to treat and per protocol effects will be estimated with logistic regression models. Furthermore, we will analyze 365-day readmission rates, time to first psychiatric hospitalization within 365 days, total number of psychiatric hospitalizations within 365 days, and cumulative number of days in hospital due to psychiatric admissions within 365 days as secondary outcomes.

**Conclusion:**

Our study will evaluate the effectiveness of sociotherapy as a transitional intervention following psychiatric hospitalization. By employing the Target Trial Emulation framework, we aim to generate robust, real-world evidence that can guide both clinical practice and policy-making, ultimately contributing to improved continuity of care for psychiatric patients.

**Trial Registration:**

Trial registration: DRKS, ISRCTNDRKS00035987. Registration on 17 February 2025—Retrospectively registered, https://drks.de/register/de/trial/DRKS00035987

**Supplementary Information:**

The online version contains supplementary material available at 10.1186/s12913-025-13137-2.

## Contributions to the literature

- The present study addresses the critical evidence gap on the effectiveness of sociotherapy, an transitional intervention currently embedded in German legislation and clinical practice.
- The study protocol outlines a Target Trial Emulation (TTE), which is a framework to evaluate causal effects based on observational real-world data. By applying this rigorous approach, we aim to generate robust evidence regarding the effectiveness of sociotherapy as a discharge management (DM) measure.
- When applicable, the findings will aid practitioners and policymakers in guiding clinical decisions and shaping policy, fostering evidence-based decision-making.

## Background

Historically, the German health care system was characterized by a strict division between inpatient and outpatient care, leading to a number of challenges in ensuring the continuity of care for patients following hospitalization [1]. To address these challenges, the German Social Code was amended in 2017 to establish mandatory standards for hospital discharge management (DM) with the aim of improving the transition from inpatient to outpatient care. Under this reform, hospitals are now authorized to prescribe medication, remedies and aids, as well as special services including sociotherapy, home care, specialized outpatient palliative care, digital health applications and transportation services. Hospitals may also certify the incapacity to work if needed and are obliged to provide a discharge document including a medication plan. Since this policy reform was introducedto facilitate the transition from inpatient to outpatient care, the duration of any measures is limited to seven days after discharge. If necessary, the measures may be continued by outpatient providers. Hospitals are mandated to initiate interprofessional discharge planning as early as possible to enhance continuity of care. This paradigm shift in the implementation of DM was enacted through a framework agreement between the National Association of Statutory Health Insurance Funds (GKV), the National Association of Statutory Health Insurance Physicians (KBV) and the German Hospital Federation (DKG) [2].

The main focus of the present paper is sociotherapy, an intervention that aims to support individuals with severe mental health disorders (i.e., schizophrenia, bipolar disorder or chronic depression). This patient-centered approach was included as a key measure in the 2017 German health care reform to improve DM and is especially relevant for psychiatric patients. As a form of structured case management, sociotherapy aims to improve coordination among healthcare providers involved in a patient’s treatment and support patients in utilizing needed healthcare services. Further goals are to foster social participation through the engagement of the patient’s social network and to offer crisis intervention if needed. In line with the Recovery Framework in mental health, the focus of sociotherapy does not lie on reducing psychiatric symptoms but rather emphasizes practical, social and daily life skills [3, 4]. Among other measures, sociotherapy ultimately aims to reduce or shorten psychiatric hospital stays [5]. Sociotherapy is typically provided by trained mental health professionals, including social workers, psychiatric nurses, and specialized therapists. Sociotherapy can be prescribed as a transitional DM measure, ideally starting immediately after discharge. To this end, an initial prescription can be issued by the hospital that lasts up to seven days, after which continued treatment requires a prescription from a licensed outpatient health care professional. In total, up to 120 sociotherapy sessions can be prescribed over a three-year period [6].

Sociotherapy as implemented in Germany shares similarities with various forms of case management found in other countries, particularly in the context of mental health care. For example, models such as Case Management (CS), Intensive Case Management, Critical Time Intervention (CTI), or Care Programme Approach (CPA) in the United Kingdom also emphasize coordinated, patient-centered care aimed at supporting individuals with severe mental illnesses [7]. Commonalities include involvement of the patient’s social network, and a focus on improving access to necessary health and social services. However, there are notable differences: the German sociotherapy framework is embedded in statutory health insurance, whereas similar programs elsewhere might operate under different funding mechanisms, timeframes or case load.

Although sociotherapy is covered by German health insurance funds for patients with severe mental health disorders, its effectiveness remains largely unexamined. This study protocol outlines a Target Trial Emulation (TTE) designed to evaluate the impact of sociotherapy as a transitional intervention on the 30-day psychiatric readmission rate. The TTE framework is particularly well suited for this purpose as it bridges the gap between randomized controlled trials (RCTs) and observational research [8, 9]. Conducting an RCT for an intervention already established by law would be challenging due to financial and logistical constraints. By systematically addressing potential biases, the TTE allows us to mimic a RCT using real-world data and gain robust evidence regarding the role of sociotherapy in provision of care in psychiatric patients.

### Design

The aim of this study protocol is to describe the emulation of a target trial designed to estimate the causal effect of sociotherapy as a DM measure using observational data from insurance claims records. Following the principles of TTE, we define the hypothetical RCT that we seek to emulate [8, 9]. Based on the primary outcome of interest (30-day readmissions), the sample size for the target trial was calculated. We then provide a detailed description of the TTE using observational data, covering key aspects such as included variables, eligibility criteria, treatment strategies, assignment procedure, follow-up period and outcomes definitions (for a comparison between hypothetical target trial and emulation see Table 1). And finally, we outline the statistical analysis strategy for the TTE.

**Table 1:** Target Trial Emulation

	**TARGET TRIAL**	**E2-PSY TRIAL EMULATION**
Eligibily Critera	Inclusion criteria • Adults aged 18 years or over • Patients with a psychiatric treatment in an inpatient, semi-inpatient or inpatient equivalent setting Exclusion criteria • Previous sociotherapy	Inclusion criteria • • Adults aged 18 years or over • Patients with a psychiatric treatment in an inpatient, semi-inpatient or inpatient equivalent setting (PEPP classification PO, PP, PA, TP, or QA) Exclusion criteria • Previous sociotherapy within 365 days before the last day of the index event • No continuous health insurance throughout the study period • Personnal of the insurance provider supplying the data • Existing data protection notice • Existing objection to use of healthcare records for research purposes
Treatment Strategies	Intervention group • Prescription of sociotherapy as acomponents of discharge management (last day of the index event) Control group • No prescription of sociotherapy as a componentof discharge management (last day of the index event)	Intervention group • Prescription of sociotherapy as a components of discharge management (last day of the index event) Control group • No prescription of sociotherapy as a component of discharge management (last day of the index event)
Assignment procedures	Participants will be randomly assigned to either treatment armon the last day of the index event. Patients will be aware of the treatment armthey have been assigned to	Quasi-randomization will be archived through a propensity score-based method, either propensity score matching or inverse probability weighting
Follow-Up Period	Starts at randomization and ends 30 days/365 days after discharge of the index event, death, or loss to follow-up	Starts at the last day of the index event and ends 30 days/365 days after discharge of the index event, death, or loss to follow-up
Outcome	Primary outcome • Readmission to hospital for psychiatric treatment within 30 days after discharge Secondary outcomes • Readmission to hospital for psychiatric treatment within 365 days after discharge • Time to first psychiatric rehospitalization • Total number of rehospitalizations within 365 days after discharge • Cumulative number of days spent in the hospital due to psychiatric admissions during 365 days after discharge	Primary outcome • Readmission to hospital for psychiatric treatment within 30 days after discharge Secondary outcomes • Readmission to hospital for psychiatric treatment within 365 days after discharge • Time to first psychiatric rehospitalization • Total number of rehospitalizations within 365 days after discharge • Cumulative number of days spent in the hospital due to psychiatric admissions during 365 days after discharge
Causal Contrasts Of Interest	• Intention-To-Treat (ITT): The ITT evaluates the average treatment effect among the treated (ATT), regardless of whether the patients adhered to the treatment • Per-Protocol (PPA): The PPA evaluates the average treatment effect among the treated (ATT), accounting for the non-adherence to the sociotherapy treatment in the intervention group, and usage of sociotherapy in the control group due to prescriptions of sociotherapy unrelated to the assignment procedure	• Intention-To-Treat (ITT): The ITT evaluates the average treatment effect among the treated (ATT), regardless of whether the patients adhered to the treatment • Per-Protocol (PPA): The PPA evaluates the average treatment effect among the treated (ATT), accounting for the non-adherence to the sociotherapy treatment in the intervention group, and usage of sociotherapy in the control group due to prescriptions of sociotherapy unrelated to the assignment procedure
Analysis Plan	• Logistic regression models for the primary outcome readmission to hospital for psychiatric treatment within 30 days after discharge and the secondary outcome readmission to hospital for psychiatric treatment within 365 days after discharge • Cumulative incidence curves and a log-rank test, as well as Cox-regression models for the outcome time to first psychiatric treatment • Poisson Regression (or negative binominal regression) for the outcomes total number of hospitalizations within 365 days after discharge and cumulative number of days spent in the hospital due to psychiatric admissions during 365 days after discharge	• Logistic regression models for the primary outcome readmission to hospital for psychiatric treatment within 30 days after discharge and the secondary outcome readmission to hospital for psychiatric treatment within 365 days after discharge • Cumulative incidence curves and a log-rank test, as well as Cox-regression models for the outcome time to first psychiatric treatment • Poisson Regression (or negative binominal regression) for the outcomes total number of hospitalizations within 365 days after discharge and cumulative number of days spent in the hospital due to psychiatric admissions during 365 days after discharge

### Sample size calculation

The required sample size for thetarget trial was determined based on the primary outcome of interest: the 30-day hospital readmission rate. To achieve sufficient statistical power (80%) to detect a minimal clinically relevant difference in the 30-day readmission rates (effect size = 0.3) between participants receiving sociotherapy and those receiving standard care, we conducted a priori power calculations (Appendix 1). Since the required sample size depends not only on the effect size but also on the outcome rate in the non-exposed group– a figure currently unknown– we calculated a range of sample sizes across plausible outcome rates [10]. Assumingan effect size of 0.3, the necessary sample size is N = 449 when the baseline readmission rate is 10%, and decreases to N = 329 when the baseline rate increases to 20%. If the required sample size based on the outcome rate in the non-exposed group (see Assignment Procedure) is not reached in our dataset, the TTE will be discontinued, and the journal will be notified. A detailed explanation outlining the reasons for the termination will be provided to ensure transparency and maintain the integrity of the study’s reporting. Exploratory analysis may be conducted.

### Trial emulation

#### Data

This study is based on insurance claims data of the German Techniker Krankenkasse (TK). TK is a German statutory health care insurance provider with a market share of approximately 15.3%. In the present study, we will use TK claims data for the years 2021 to 2024 of each individual fulfilling the eligibility criteria in 2022 to 2023 [11].

#### Eligibility criteria

The inclusion criteria for the study require participants to have received at least one psychiatric or psychosomatic treatment^1^ in an inpatient, semi-inpatient, or inpatient equivalent setting between January 2022 to December 2023, under the Psychiatric and Psychosomatic Compensation System (German Akronym: PEPP). In a PEPP number, the first two positions define the structural category, while the subsequent positions offer further specification. Included PEPP structural categories are PO (particularly complex cases), PP (psychosomatics), and PA (psychiatry) for inpatient treatment. For semi-inpatient and inpatient equivalent treatments, the relevant categories are TP and QA (both psychiatry). Not included are the categories for child and youth psychiatry and psychotherapy (codes PK, TK, and QK), in each care setting. Additionally, participants must have reached the age of 18 by the time of the index event. Since a structured DM is required by law we do not include it as an inclusion criterion.

The exclusion criteria for the study are: a previous inpatient or outpatient prescription of sociotherapy within 365 days before the last day of the index event. Additionally, we will exclude individuals who did not maintain continuous insurance coverage throughout the study period including baseline and follow-up period (January 2021– December 2024); employees of TK; individuals who have a registered data protection notice; and those who have explicitly objected to the use of their healthcare records for research purposes.

### Treatment strategies

#### Intervention group

The intervention group receives a prescription for sociotherapy as a component of the structured DM.

#### Control group

The control group receives standard care, which includes regular DM following psychiatric treatment but does not involve a prescription for sociotherapy.

### Assignment procedures

To address potential confounding in the observational data we estimate propensity scores based on the probability of receiving sociotherapy. These scores will be calculated using logistic regression controlling for key characteristics of the index event, other prescribed DM measures, medical history and sociodemographic characteristics (for a detailed description see Table 2). Afterwards the propensity scores will be applied by either propensity score matching (PSM) or inverse probability weighting (IPW) depending on the distribution of the scores. Balance diagnostics such as standardized mean differences will be conducted and all steps of the procedure including the applied methods will be reported.

### Follow-up period

We follow up with participants for 30 days after discharge to assess the primary outcome, and for up to 365 days for secondary outcomes, or until death—whichever occurs first. During the follow-up period, we will evaluate the continuation of sociotherapy prescriptions as a component of a structured DM, as well as any new prescriptions issued in the outpatient setting.

### Outcomes

The primary outcome is the average treatment effect among the treated (ATT) on the 30-day readmission rate to a psychiatric hospital. A readmission is defined as a subsequent admission to an inpatient setting for patients who were previously discharged from an inpatient setting. For patients who received the index treatment in an inpatient-equivalent setting, a readmission includes admission to inpatient, or inpatient-equivalent setting. For patients who received the index treatment in a semi-inpatient setting, a readmission includes admission to any of these levels of care: inpatient, semi-inpatient, or inpatient-equivalent. However, a transfer between these settings is not considered a readmission but rather a continuation of the index event, provided that the transfer occurs on the same day. In addition to the primary outcome, we define the following secondary outcomes: the ATT on 365-day readmission rate for psychiatric hospitalization, the ATT on time to first psychiatric readmission, the ATT on total number of psychiatric hospitalizations within a 365-day period, and the cumulative number of days spent in the hospital due to psychiatric admissions during this time frame.

### Statistical analysis

#### Descriptives

Descriptive statistics will be used to summarize the baseline characteristics of participants prior to and after PSM. Continuous variables will be reported as means with standard deviations or medians with interquartile ranges, depending on their distribution, while categorical variables will be presented as frequencies and percentages. To assess baseline balance between the groups after PSM, the Standardized Mean Difference (SMD) will be calculated for all variables, with a SMD of less than 0.1 indicating a good balance.

#### Intention-to-Treat Analysis (ITT)

All patients assigned to the study population within the PSMprocedure will be included in the ITT. Patients will be analyzed according to the group to which they were assigned.

#### Per-Protocol Analysis (PPA)

The per-protocol population will include only those patients who were assigned to the study population within the PSM procedure and adhere to the study protocol. For the intervention group adherence will be defined as the utilization of least one sociotherapy session prescribed as a DM measure. For the control group adherence will be defined as standard care. However, sociotherapy sessions prescribed in any other setting will be defined as a violation of the protocol for this group.

### Analyses of primary and secondary outcomes

The primary outcome will be analyzed with logistic regression models to evaluate the association between sociotherapy and the 30-day readmission rate. This model will be adjusted for potential confounders as described below. Both ITT and PPA will use the same statistical models for the analysis. However, since the magnitude of the odds ratios estimated in logistic regression model cannot be compared across models, we can only compare whether the effects are positive or negative [12, 13].

For the secondary outcome 365-day readmission to psychiatric hospitalization, we will apply the same procedure as for the primary outcome 30-day readmission. Regarding time to first psychiatric readmission, we will generate cumulative incidence curves to illustrate the time to readmission for participants in both the intervention and control group. The log-rank test will be employed to compare the distributions. Further, we will use Cox-regression models to account for confounders. The secondary outcome total number of psychiatric hospitalizations within a 365-day period and cumulative number of days spent in the hospital due to psychiatric admissions we will apply Poisson regression models. In cases where overdispersion is detected, we will instead use negative binomial regression models. If a high prevalence of zero counts is observed, we will address this by applying zero-inflated Poisson or zero-inflated negative binomial regression models. Both ITT and PPA will be applied to all secondary outcomes.

### Confounders

In all multivariate analyses, we will adjust for key characteristics of the index event, including the PEPP category, the length of the index event, diagnoses of index event (dementia, substance abuse, schizophrenia and schizoaffective disorder, bipolar disorder and manic episodes, major depressive disorder, post-traumatic stress disorder, and personality disorder), the necessity for intensive treatment (OPS-Code 9–61), whether the admission was an emergency admission, other prescribed or certified DM measures (medication, remedies and aids, home care, outpatient intensive care specialized outpatient palliative care, patient transportation, digital health applications, and incapacity certificate). We will also control for participants' medical history prior to the index hospitalization, as such previous psychiatric hospitalizations, comorbidities in form of the Elixhauser Comorbidity Index [14] and care level. As for demographic variables, we will include age, sex, population density at the place of residence and the German Index of Socioeconomic Deprivation [15] (Table 2).

**Table 2:** Variables

Category	Variable	Operationalization
Exposure	Sociotherapy	Prescription of Sociotherapy as a DM measure (ITT) / Prescription of Sociotherapy as a DM and utilization of at least one sociotherapy session (PPA)
Index event	PEPP	PO/PP/PA/TP/QA
	Length of index event	Number of days of index event
	Dementia	F00.x– F03.x, G30.x
	Substance abuse	F10.2, F11.2, F12.2, F13.2, F14.2, F15.2, F16.2, F17.2, F18.2, F19.2
	Schizophrenia & Schizoaffective disorder	F20.x, F21.x; F25.x
	Bipolar disorder & Manic episodes	F30.1– F30.2, F31.1– F31.2, F31.4- F31.6
	Major depressive disorder	F32.2– F32.3; F33.2– F33.3
	Post-traumatic stress disorder	F43.1
	Personality disorder	F60.x– F61.x
	Intensive treatment	OPS 9–61
	Emergency admission	Reason for admission
Discharge management measures (presciptions and certificates)	Medication	
	Remedies and aids	
	Home care	
	Outpatient intensive care	
	Specialized outpatient palliative care	
	Patient transportation	
	Digital health applications	
	Incapacity certificate	
Medical history	Elixhauser Comorbidity Index [16]	Based on all available diagnoses twelve months prior to the index event
	Number of psychiatric hospital days	Hospital stays with PEPP categories PO, PP, PA, TP & QA based on all available data twelve months prior to the index event
	Care level	Based on care level on the last day prior to the index event
Sociodemographic	Age	
	Sex	
	Population density at place of residence	NUTS 3 (Destatis data)
	German Index of Socioeconomic Deprivation (GISD) at place of residence	NUTS 3 (GISD data)

## Discussion

By employing the TTE framework, we adopt a systematic approach that minimizes the risk of bias when analyzing real-world data, thereby enhancing the reliability and validity of our findings. If feasible, our study will be among the first to provide robust evidence on the effectiveness of sociotherapy for reducing readmission rates among psychiatric inpatients. However, the insurance claims data used in this study provide limited and potentially less reliable information on key social determinants, such as social support, family or employment status. As a result, we are unable to account for these potential confounders, which may influence the primary and secondary outcomes. Furthermore, the reduction in psychiatric readmissions or the shortening of psychiatric hospital stays are not the only potential benefits of sociotherapy. Our study does not evaluate the impact of sociotherapy impact on the utilization of outpatient healthcare services and better coordination between healthcare providers, nor does it consider broader outcomes such as patient motivation, social participation, or the engagement of the patient’s social network, all of which are key goals of sociotherapy.

## Conclusion

In conclusion, our study aims to provide robust evidence regarding the effectiveness of sociotherapy prescriptions as a DM component on psychiatric readmissions. By employing the TTE framework, we aim to minimize bias and enhance the reliability of our findings which are derived from real-world data. The study addresses a critical lack of evidence for a treatment which is codified in German law and covered by German health insurances. If sociotherapy proves to be effective on the reduction of psychiatric readmissions, the findings could support its broader implementation and integration into routine care. Conversely, if no significant effect is observed, the results could spark a renewed discussion about the treatment’s role and barriers—ranging from exploring other potential beneficial outcomes, such as improved social participation and patient engagement, exploring potential barriers of implementation especially in regard to a fast provision after hospital discharge versus long, bureaucratic processes, to reconsidering its inclusion in health insurance coverage.

## Supplementary Information

Supplementary Material 1.

Supplementary Material 2.

## Data Availability

No datasets were generated or analyzed during the current study.

## Abbreviations

DM: Discharge Management
GKV: National Association of Statutory Health Insurance Funds
KBV: National Association of Statutory Health Insurance Physicians
DKG: German Hospital Federation
CS: Case Management
CTI: Critical Time Intervention
CPA: Care Programme Approach
TTE: Target Trial Emulation
RCT: Randomized Controlled Trials
TK: Techniker Krankenkasse
PEPP: Psychiatric and Psychosomatic Compensation System
PSM: Propensity Score Matching
IPW: Inverse Probability Weighting
ATT: Average Treatment Effect Among the Treated
ITT: Intention-to-Treat Analysis
PPA: Per-Protocol Analysis
SMD: Standardized Mean Difference
NUTS: Nomenclature des Unités territoriales statistiques

## Footnotes

1 In Germany, there is a formal distinction between psychiatry and psychosomatic medicine in terms of inpatient care structures and reimbursement systems, which is uncommon in many other countries. While both fields may treat patients with psychiatric (F-coded) diagnoses, this study uses the term'psychiatry'throughout for simplicity and to align with international conventions, as the distinction does not substantially affect the study's focus or findings.
