# Supplementary material for: Evaluating the effectiveness of sociotherapy following psychiatric hospitalization: a target trial emulation protocol using German statutory health insurance data
Source: BMC Health Serv Res. 2025 Jul 31;25:1012. doi: 10.1186/s12913-025-13137-2 (PMC12315314; doi:10.1186/s12913-025-13137-2)
Supplement: Supplementary file 1 — Supplementary Material 1. [file 12913_2025_13137_MOESM1_ESM.docx]

E2-PSY Target Trial Emulation

Sample Size Calculation

Raphael Kohl and Julie O´Sullivan

# Introduction

Sociotherapy is a form of mental health treatment designed to support patients with severe mental health disorders. As a form of structured case management, sociotherapy aims to improve coordination among healthcare providers involved in a patient’s treatment and support patients in utilizing needed healthcare services. Further goals are to foster social participation through the engagement of the patient’s social network and to offer crisis intervention if needed. Among others sociotherapy aims to reduce or shorten psychiatric hospital stays. Sociotherapy can be prescribed as a discharge management (DM) measure following a psychiatric hospital stay.

While sociotherapy is covered as a DM measurement for patients with severe mental health disorders by German health insurance funds,[1,2] evidence regarding its effectiveness remains limited. Our Target Trial Emulation (TTE) seeks to evaluate the impact of sociotherapy on 30-day readmission rates following discharge as the primary outcome.

Here, we calculate the sample size required to achieve sufficient statistical power to detect a meaningful effect. Since our primary analysis involves logistic regression with a focus on calculating odds ratios, this entails accounting for the baseline 30-day readmission rate.

# Methods

All analyses were performed in R version 4.2.2.[3] Sample sizes were calculated using the R package {epiR}.[4] Other used packages are {dyply}, and {ggplot2}.[5,6]

# Outcome of interest

We define the 30-day readmission rate as our primary outcome and use a plausible range of 10% to 20% for the control group in our sample size calculation. This range is informed by a recent meta-analysis reporting a 16% readmission rate (95% CI: 13-20%) [7] and a separate study, which reports re-hospitalization rates of psychiatric patients to be 15.5%.[8]

# Sample size calculation

To determine the minimal required sample size, it is crucial to estimate the odds Ratio (OR), which depends not only on the effect size but also on the outcome rate in the non-exposed group.[9] To adress this, we employed the following function to calculate the OR, capturing its dependence on these parameters. [Figure 1](#fig-es_or) illustrates the relationship between the OR and the effect size.

cohen_to_or <- function(p0,d){

 if (p0 <= 0|| p0 >= 1) {
 stop("The value of p must be between 0 and 1")
 }

 z0 <- qnorm(p0) # calculate the standard normal
 # deviation for p0
 z1 <- z0 + d # calculate the standard normal
 # deviation for p1 (including d)
 p1 <- pnorm(z1) # calculate the rate of the outcome
 # fo intrest in the exposed group
 or <- (p1*(1-p0))/(p0*(1-p1)) # calculate the odds ratio

 result <- list(
 p0 = p0,
 z0 = z0,
 p1 = p1,
 z1 = z1,
 OR = or)
 return(result)
}

| 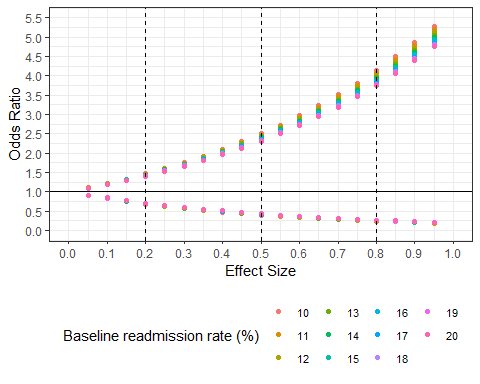  Figure 1: Odds Ratios at different effect sizes |
| --- |

## Results

As illustrated in [Figure 2](#fig-es_30), the required sample size is influenced by both the baseline readmission rate and the effect size. For an effect size of 0.3, the necessary sample size is 449 when the baseline readmission rate is 10%, and decreases to 329 when the baseline rate increases to 20%. For a larger effect size of 0.5, the required sample size drop significantly, with 152 needed at a baseline readmission rate of 10%, and to 115 at 20%.

| 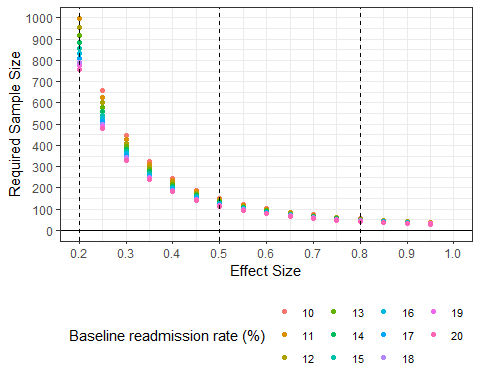  Figure 2: Required Sample Size Based on Different Effect Sizes |
| --- |

# Conclusion

We define an effect size of 0.3 as the minimal clinically relevant impact on our outcome of interest. Based on our analysis, achieving this effect size requires a sample size ranging from 329 to 449 patients per group, depending on the baseline readmission rate. To ensure the study’s validity, we define the shortfall in meeting these sample sizes as an abort criterion for our target trial emulation.

# References

[1] Gühne U, Weinmann S, Riedel-Heller SG, Becker T, editors. S3-Leitlinie Psychosoziale Therapien bei schweren psychischen Erkrankungen: S3-Praxisleitlinien in Psychiatrie und Psychotherapie. Berlin, Heidelberg: Springer Berlin Heidelberg; 2019. <https://doi.org/10.1007/978-3-662-58284-8>.

[2] G-BA. Gemeinsamer Bundesausschuss. Richtlinie des Gemeinsamen Bundesausschusses Über die Durchführung von Soziotherapie in der vertragsärztlichen Versorgung (Soziotherapie-Richtlinie/ ST-RL) in der Neufassung vom 22. Januar 2015; 2015.

[3] R Core Team. R: A language and environment for statistical computing. Vienna, Austria: R Foundation for Statistical Computing; 2022.

[4] Stevenson M, Sergeant E. epiR: Tools for the Analysis of Epidemiological Data 2024.

[5] Wickham H, François R, Henry L, Müller K, Vaughan D. Dplyr: A grammar of data manipulation. 2023.

[6] Wickham H. Ggplot2: Elegant graphics for data analysis. Springer-Verlag New York; 2016.

[7] Muhammad N, Talpur S, Sangroula N, Washdave F. Independent Predictors of 30-Day Readmission to Acute Psychiatric Wards in Patients With Mental Disorders: A Systematic Review and Meta-Analysis. Cureus 2023. <https://doi.org/10.7759/cureus.42490>.

[8] Katschnig H, Straßmayr C, Endel F, Berger M, Zauner G, Kalseth J, et al. Using national electronic health care registries for comparing the risk of psychiatric re-hospitalisation in six European countries: Opportunities and limitations. Health Policy 2019;123:1028–35. <https://doi.org/10.1016/j.healthpol.2019.07.006>.

[9] Chen H, Cohen P, Chen S. How Big is a Big Odds Ratio? Interpreting the Magnitudes of Odds Ratios in Epidemiological Studies. Communications in Statistics - Simulation and Computation 2010;39:860–4. <https://doi.org/10.1080/03610911003650383>.
